# Changes in cortical grey matter volume with Cognitive Orientation to daily Occupational Performance intervention in children with developmental coordination disorder

**DOI:** 10.3389/fnhum.2024.1316117

**Published:** 2024-05-22

**Authors:** Myrah Anum Malik, Alexander Mark Weber, Donna Lang, Tamara Vanderwal, Jill G. Zwicker

**Affiliations:** ^1^Graduate Programs in Rehabilitation Science, University of British Columbia, Vancouver, BC, Canada; ^2^Brain, Behaviour, and Development Theme, BC Children’s Hospital Research Institute, Vancouver, BC, Canada; ^3^Department of Pediatrics, University of British Columbia, Vancouver, BC, Canada; ^4^Department of Radiology, University of British Columbia, Vancouver, BC, Canada; ^5^Department of Psychiatry, University of British Columbia, Vancouver, BC, Canada; ^6^Department of Occupational Science and Occupational Therapy, University of British Columbia, Vancouver, BC, Canada

**Keywords:** developmental coordinator disorder, motor skills disorder, children, CO-OP, rehabilitation, MRI, brain structure, voxel-based morphometry

## Abstract

**Introduction:**

Cognitive Orientation to daily Occupational Performance (CO-OP) is a cognitive-based, task-specific intervention recommended for children with developmental coordination disorder (DCD). We recently showed structural and functional brain changes after CO-OP, including increased cerebellar grey matter. This study aimed to determine whether CO-OP intervention induced changes in cortical grey matter volume in children with DCD, and if these changes were associated with improvements in motor performance and movement quality.

**Methods:**

This study is part of a randomized waitlist-control trial (ClinicalTrials.gov ID: NCT02597751). Children with DCD (*N* = 78) were randomized to either a treatment or waitlist group and underwent three MRIs over 6 months. The treatment group received intervention (once weekly for 10 weeks) between the first and second scan; the waitlist group received intervention between the second and third scan. Cortical grey matter volume was measured using voxel-based morphometry (VBM). Behavioral outcome measures included the Performance Quality Rating Scale (PQRS) and Bruininks-Oseretsky Test of Motor Proficiency-2 (BOT-2). Of the 78 children, 58 were excluded (mostly due to insufficient data quality), leaving a final *N* = 20 for analyses. Due to the small sample size, we combined both groups to examine treatment effects. Cortical grey matter volume differences were assessed using a repeated measures ANOVA, controlling for total intracranial volume. Regression analyses examined the relationship of grey matter volume changes to BOT-2 (motor performance) and PQRS (movement quality).

**Results:**

After CO-OP, children had significantly decreased grey matter in the right superior frontal gyrus and middle/posterior cingulate gyri. We found no significant associations of grey matter volume changes with PQRS or BOT-2 scores.

**Conclusion:**

Decreased cortical grey matter volume generally reflects greater brain maturity. Decreases in grey matter volume after CO-OP intervention were in regions associated with self-regulation and motor control, consistent with our other studies. Decreased grey matter volume may be due to focal increases in synaptic pruning, perhaps as a result of strengthening networks in the brain via the repeated learning and actions in therapy. Findings from this study add to the growing body of literature demonstrating positive neuroplastic changes in the brain after CO-OP intervention.

## 1 Introduction

Developmental coordination disorder (DCD) is classified as a neurodevelopmental disorder in the Diagnostic and Statistical Manual 5th edition (DSM-5). This motor disorder affects approximately 450,000 Canadian school-aged children (American Psychiatric Association, 2013; Statistics Canada, 2021). The related gross and fine motor difficulties affect important childhood activities such as tying shoelaces, printing, or riding a bicycle (Kirby and Sugden, 2007; Zwicker et al., 2012; Blank et al., 2019). Early intervention is important, as children with DCD typically continue to experience motor difficulties well into adolescence and adulthood if adequate intervention is not provided throughout childhood (Kirby et al., 2013).

Traditionally, interventions have been process-oriented and focused on addressing the sensorimotor dysfunction that was thought to contribute to their motor impairments (Polatajko et al., 2001; Mandich et al., 2002; Polatajko and Mandich, 2004). Newer approaches leverage current theories of cognitive and motor learning and advocate for problem-solving focused intervention (Sugden, 2007). One such intervention, the Cognitive Orientation approach to daily Occupational Performance (CO-OP), was developed by occupational therapists in Canada (Polatajko et al., 2001). This task-specific intervention is a cognitive-based, problem-solving approach that uses verbal mediation and identifies strategies to support motor skill acquisition (Polatajko et al., 2001). Several systematic reviews have been conducted that further demonstrate the effectiveness of CO-OP intervention for children with DCD (Smits-Engelsman et al., 2012, 2018; Scammell et al., 2016; Yu et al., 2018), making CO-OP one of the recommended treatments in the international clinical practice guidelines for DCD (Blank et al., 2019).

While CO-OP has been deemed effective, the underlying mechanisms or neural bases for clinical improvements were unknown. Our research group recently used magnetic resonance imaging (MRI) to investigate brain changes associated with CO-OP intervention. In a study that focused on the cerebellum, we showed increases in grey matter volume in the brainstem and in cognitive (right crus II) and motor regions (right and left lobule VIIIb and lobule IX) of the cerebellum following the intervention (Gill et al., 2022). Improvements in actual movement performance predicted the increases in cerebellar grey matter volume. In addition, increased functional connectivity in the default mode network and right anterior cingulate cortex were observed after CO-OP intervention (Izadi-Najafabadi et al., 2022b), as well as improved white matter microstructure in several regions, including the bilateral anterior thalamic radiations, bilateral sensorimotor tracts, bilateral cingulum, and corpus callosum (Izadi-Najafabadi and Zwicker, 2021). These brain regions are associated with attention, self-regulation, motor planning, and inter-hemispheric communication (Izadi-Najafabadi and Zwicker, 2021).

Cortical brain structures undergo developmental changes during childhood (Giedd et al., 1999; Wierenga et al., 2014; Gilmore et al., 2018), and some therapies (e.g., behavioral, medications) have been associated with changes in cortical volume in children with neurodevelopmental disorders (Spencer et al., 2013; Sterling et al., 2013). To date, it is unknown whether CO-OP induces neuroplastic change in cortical brain structure. The aims of this study were to determine: (1) whether CO-OP intervention induces changes in cortical grey matter volume of children with DCD; and (2) if any grey matter volume changes are associated with improvements in motor performance and movement quality. We hypothesized that following CO-OP intervention, we would find: (1) increased cortical grey matter volume in regions of the brain associated in coordinating motor and executive functioning skills (i.e., parietal and frontal lobe); and (2) positive associations between changes in grey matter volume, motor performance, and movement quality improvements.

## 2 Materials and methods

### 2.1 Study design

**Figure 1:**
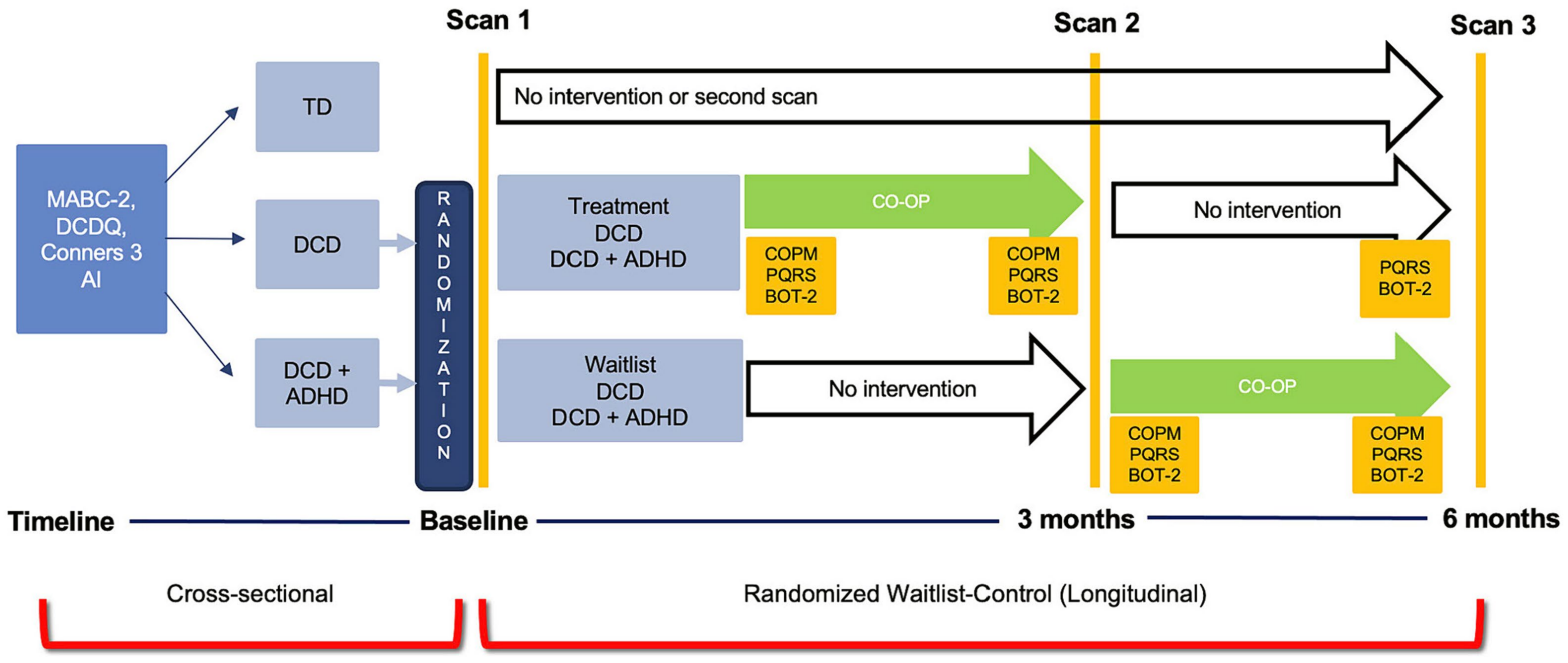
Study design for the cross-sectional and randomized waitlist-control trial for the Zwicker Lab DCD imaging study.

This study was a part of a randomized waitlist-control trial using multiple neuroimaging modalities to assess brain changes with CO-OP intervention (ClinicalTrials.gov ID: NCT02597751). For the purpose of this study, structural MRI data were collected before and after CO-OP intervention to investigate changes in grey matter volume in children with DCD and in children with DCD and co-occurring ADHD (DCD + ADHD). Participants received treatment either after the first MRI (treatment group) or after a 3-month waiting period (waitlist group). A statistician randomized participants using computer-generated sequential blocks of 4 to 6. The randomization codes, either treatment or waitlist, were sealed in opaque envelopes until study enrollment. After screening and recruitment, all parents or legal guardians provided written consent and children assented to participate in the study. The study design (randomized waitlist-control trial) for the purpose of this paper is shown in Figure 1. All aspects of the study were approved by the Children’s and Women’s Health Centre/University of British Columbia Research Ethics Board (#H14-00397).

### 2.2 Participants

A convenience sampling strategy was used to recruit participants between September 2014 to July 2019. The following sources were used to recruit participants for the intervention: (1) Dr. Zwicker’s research-integrated DCD clinic at Sunny Hill Health Centre for Children; (2) BC Children’s Hospital ADHD Clinic; (3) from caseloads of occupational and/or physical therapists from Sunny Hill and the Vancouver Regional Pediatric Team who service schools in the Vancouver and surrounding districts; and (4) the community. Community recruitment was done by using bulletin boards at BC Children’s Hospital, UBC, and Vancouver schools. TD children were recruited through advertisements in Vancouver schools and community centres, and by word-of-mouth.

Inclusion criteria were based on the DSM-5 diagnostic criteria (American Psychiatric Association, 2013) and international clinical practice recommendations for DCD diagnosis (Blank et al., 2019) as follows: (1) scores ≤16th percentile on the MABC-2 (Henderson et al., 2007); (2) score in the suspected or indicative range on the Developmental Coordination Disorder Questionnaire (DCDQ) (Wilson and Crawford, 2007); (3) parent-reported motor difficulties from a young age; and (4) no other medical condition that could explain motor difficulties as per parent-reports, clinical reports and/or medical examination. Participants were excluded if they were born preterm (gestational age week <37 weeks) or diagnosed with other neurodevelopmental disorders, such as autism spectrum disorder.

The control group (TD children) included children 8–12-years old with no history of motor difficulties and a MABC-2 score ≥25th percentile. Exclusion criteria included being born preterm (gestation week <37 weeks) or diagnosed with any other neurodevelopmental disorder, such as autism spectrum disorder. Children assigned to the TD group were excluded if they were diagnosed with ADHD.

### 2.3 Procedure

Prior to enrollment, all participants were administered the Movement Assessment Battery for Children (MABC-2) to quantify the level of motor impairment (Henderson et al., 2007) and whether the participants met the inclusion criteria for the study. In addition, the DCDQ parent-completed questionnaire was used to identify motor impairments of the participants in comparison to their peers (Wilson and Crawford, 2007). Lastly, the Conners 3 ADHD Index parent form was used to assess for attentional performance (Conners, 2009). Scores 70 and above are considered to be clinically significant (poorer attentional performance indicates greater attentional difficulties).

Both scanning and intervention took place at BC Children Hospital Research Institute. All children participated in a magnetic resonance imaging (MRI) safety screening and were informed about the MRI procedure. An MRI simulator session was done to familiarize the children with the scanning environment (noise, confined space, and head coil). They were also provided with strategies from the research team to help reduce potential for anxiety. The simulator session helped to answer the child’s and/or parent’s questions and inform the research team about the child’s ability to remain still in the MRI scan, as the scans are sensitive to motion (Zaitsev et al., 2015).

After the first MRI session, children were randomly assigned to either the treatment or waitlist group, so that the research team was blinded to group allocation until after the first MRI. Children in both groups had three MRI sessions: (1) scan 1 occurred at enrollment; (2) scan 2 was conducted 3 months after the first scan (to measure treatment effect in the treatment group and maturation in the waitlist group); and (3) scan 3 occurred 6 months after the first scan (to assess follow-up 3 months after intervention in the treatment group and to measure the treatment effect of the waitlist group). Following the first MRI session, children in the treatment group received CO-OP intervention (led by an occupational therapist) once weekly for 10 weeks; they then had a post-intervention scan, and another follow-up scan 3 months later. Children in the waitlist group waited for 3 months for their second MRI and then began CO-OP intervention for 10 weeks; they had a third MRI after intervention (Figure 1). Caregivers were encouraged to attend treatment sessions so that therapists could instruct them how to facilitate strategy use between treatment sessions. Prior to intervention, children selected three functional motor goals (e.g., printing, tying shoes, performing sport-related movements) that they wanted to achieve over the 10 weeks of therapy; each session lasted an hour. Outcomes measures included the Canadian Occupational Performance Measure (COPM) (Law et al., 2014), Performance Quality Rating Scale (PQRS) (Martini et al., 2014), and Bruininks-Oseretsky Test of Motor Proficiency-2 (BOT-2) (Bruininks and Bruininks, 2005). The COPM and BOT-2 were administered by an occupational therapist not involved in the intervention. The PQRS was video recorded by the treating therapist before and after intervention but was scored by the assessing therapist who was blinded to pre-test/post-test status.

### 2.4 Clinical outcome measures

#### 2.4.1 Canadian Occupational Performance Measure

The COPM (Law et al., 2014) is a client-centered questionnaire that was administered by an occupational therapist before and after the completion of the 10-week CO-OP intervention. It allows the individual to rate their performance and satisfaction for each of their self-chosen goals on a scale of 1 to 10, where a higher score indicates increased levels of performance and satisfaction with their self-chosen goals (Law et al., 2014). A two-point change is considered clinically meaningful (Carswell et al., 2004; Law et al., 2014). The COPM is considered a valid, reliable, and responsive outcome measure (Carswell et al., 2004; Dedding et al., 2004; Eyssen et al., 2011).

#### 2.4.2 The Performance Quality Rating Scale

The PQRS is a 10-point performance rating scale to evaluate changes in observed movement quality during task performance; higher scores indicate better movement quality (Miller et al., 2001; Polatajko and Mandich, 2004). The PQRS has moderate to substantial inter-rater reliability, excellent test-retest reliability, and good internal responsiveness (Miller et al., 2001; Martini et al., 2014). Before and after CO-OP intervention, children were video-recorded performing their chosen goals. An occupational therapist who was not engaged in the delivery of the intervention and was blinded to the pre/post assessment sessions scored the videos. The child’s actual performance quality was rated on a scale of 1 to 10 (1 being “cannot do the skill at all” and 10 being “does the skill very well”) (Martini et al., 2014). An increase of three points is considered clinically significant (Martini et al., 2014). The PQRS complements the COPM by measuring the actual, rather than perceived, performance of the child’s self-chosen goals.

#### 2.4.3 Bruininks-Oseretsky Test of Motor Proficiency-2

The short form of BOT-2 (Bruininks and Bruininks, 2005) was completed for this study. This short form consists of one or two items from each of the eight areas: bilateral coordination, balance, running speed and agility, strength, fine-motor precision, fine-motor integration, manual dexterity, and upper extremity coordination (Bruininks and Bruininks, 2005). The BOT-2 is a standardized, norm-referenced assessment that measures motor performance (Deitz et al., 2007), where a higher percentile scoring indicates better motor performance. This assessment is reported to have moderate to strong inter-rater/test–retest reliability (Deitz et al., 2007), excellent concurrent validity with other motor measures, and adequate construct and content validity (Slater et al., 2010). The total percentile scores of the BOT-2 short-form were used for analysis.

### 2.5 Neuroimaging measures

#### 2.5.1 MRI data acquisition

All brain images were acquired at the MRI Research Facility at BC Children’s Hospital Research Institute in Vancouver, Canada. MRI scans were obtained on a 3-Tesla General-Electric Discovery MR 750 scanner. T1-weighted 3D structural scans were acquired with the following parameters: three-dimensional spoiled gradient recalled acquisition in steady state (3D SPGR), echo time = 30 ms, repetition time = 3,000 ms, FOV = 256, matrix size = 256 × 256, flip angel = 12^°^, number of slices = 256, slice thickness = 1 mm, interleaved with no gaps (voxel size 0.9375 × 0.9375 × 1 mm). Using T1 weighted scans allows for reliable segmentation of tissues (grey matter, white matter, and cerebrospinal fluid) and permits reliable identification of underlying regions (Lerch et al., 2017).

#### 2.5.2 Image quality control

All scans were visually inspected for truncation, motion, aliasing-related and other artifacts (Krupa and Bekiesińska-Figatowska, 2015; Reuter et al., 2015). Specifically, image quality was assessed for head coverage, wrapping artifact, radiofrequency noise, signal inhomogeneity, susceptibility artifact, and ringing artifact (Reuter et al., 2015). An ordinal score was given to each image based on motion artifacts and image quality (pass, questionable, or fail) using standardized methodology (Harvard Center for Brain Science, 2014). Two trainees assessed the scans independently; the level of agreement for the categorization of each scan assessed by each trainee was 96%. Only scans that passed the final quality check from both trainees were included in the analysis. Additionally, quantitative measures of motion were calculated using the software package MRIQC (10.1371/journal.pone.0184661). In particular, we measured coefficient of joint variation (CJV), where higher values are related to the presence of heavy head motion and large intensity non-uniformity (10.3389/fninf.2016.00010). Forty-five participants with DCD with significant motion artifact or poor grey to white matter differentiation were excluded from the larger sample to produce the final dataset of 20 participants with two good quality scans before and after intervention.

Twenty-two TD participants with similar data quality artifacts were excluded, resulting in nine participants with good quality scans acquired 3 months apart.

#### 2.5.3 Voxel-based morphometry

##### 2.5.3.1 Image pre-processing

Data were converted from DICOM (Digital Imaging and Communications in Medicine) to NIfTI (Neuroimaging Informatics Technology Initiative) format using the dcm2nii tool from MRIcron.^1^ T1 images were processed using voxel-based morphometry (VBM), a computational technique that measures differences in grey matter volume through a voxel-wise comparison (Ashburner and Friston, 2000; Whitwell, 2009). VBM uses T1-weighted MRI scans and performs a voxel-by-voxel statistical analysis across each image to identify volume differences between patients and controls (Ashburner and Friston, 2000). All pre-processing and longitudinal VBM data analysis were carried out using the Computational Anatomy Tool Box (CAT12, v1742, The Structural Brain Mapping Group, Jena, Germany, http://dbm.neuro.uni-jena.de/cat12/), through Statistical Parametric Mapping 12 software (SPM12, v7771, The Wellcome Centre for Human Neuroimaging, London, United Kingdom, https://www.fil.ion.ucl.ac.uk/spm/) in MATLAB R2020b (Mathworks, Natick, Massachusetts, United States).

For image preprocessing, all T1 images were manually registered to the anterior commissure at the origin of the Montreal Neurological Institute (MNI) coordinate system (Jahn, 2019). Initially in longitudinal VBM analysis, intra-participant co-registration was performed on the pre- and post-intervention images. The co-registered images were then realigned across participants and bias-corrected with reference to the mean images computed from each participant’s pre- and post-intervention images. The images were then segmented into grey matter, white matter, and cerebrospinal fluid (CSF) with the customized pediatric tissue probability maps from Template-O-Matic Toolbox (TOM8 https://neuro-jena.github.io/software.html#tom) as an initial estimate. All images were included if their weight average Image Quality Rating (IQR) was greater than 80%, corresponding to a “good” image quality. Mean correlations between all volumes were visualized through CAT12. Volumes with a correlation below two standard deviations from the sample mean were again visually inspected for artifacts. Next, good quality pre- and post-average affine-registered white and grey matter tissue segments were extracted to construct a customized Diffeomorphic Anatomical Registration Through Exponentiated Lie Algebra (DARTEL) study-specific template registered to the MNI-International Consortium for brain Mapping (ICBM) space. This alternative to the adult-based template provided by CAT12 was used to achieve a more accurate inter-participant registration to improve the realignment of small inner structures for an overall better segmentation (Good et al., 2001; Yassa and Stark, 2009). Likewise, this additional step is similar to VBM studies done in other pediatric neurodevelopmental disorder studies that created a study-specific average template for their sample (Reynolds et al., 2017; Wang et al., 2017; Sáenz et al., 2020). The images were then normalized using an affine spatial normalization and a further modulation was applied to convert the voxel values of tissue concentration to measures of volume. Finally, the normalized grey matter maps were smoothed with an isotropic Gaussian kernel (full width at half maximum = 6 mm). Total intracranial volume (TIV) was calculated for the pre and post grey matter, white matter, and CSF images for each participant using CAT12 module “Total intracranial volume.”

##### 2.5.3.2 Computational Anatomy Toolbox

The Structural Brain Mapping Group at the University of Jena (Jena, Germany) designed the automatic and easy-to-use toolbox CAT12 as an extension to the SPM software. CAT12 follows a standard VBM analysis pipeline similar to VBM8. We used segmentation through SPM’s extension CAT12 rather than FreeSurfer or FSL as SPM produces a more robust segmentation for those with limited image quality (Fellhauer et al., 2015). This decision was further supported through a comparison to previous toolboxes, with CAT12 providing a more accurate and robust volumetric analysis (Farokhian et al., 2017) and advanced segmentation tool (Tavares et al., 2020). It has also been used in neurodevelopmental disorders that commonly co-occur with DCD (Wang et al., 2017; Mei et al., 2020; Sáenz et al., 2020) where the workflow was adapted to accommodate a pediatric population as recommended for VBM analysis.

### 2.6 Statistical analysis

#### 2.6.1 Participant characteristics

Jeffreys’s Amazing Statistics Program (JASP https://jasp-stats.org/) was used to summarize participant characteristics [age, sex, TIV, MABC-2 (motor ability), DCDQ (motor function), and Conners 3 ADHD Index (ADHD symptomatology)] and pre-post intervention outcome measures. The behavioral data for the entire cohort have been reported by Izadi-Najafabadi et al. (2022a). Here, we report motor outcome data for the sub-sample in this paper. The Wilcoxon Signed-Rank Test (non-parametric) was used to compare the before and after effect of CO-OP intervention on average COPM performance and satisfaction scores, average PQRS total actual performance scores, and BOT-2 motor percentile ranks. The alpha level was set to 0.05 with Bonferroni correction used to correct for multiple comparisons when comparing pre- post values to avoid type 1 errors.

#### 2.6.2 Longitudinal VBM statistical analysis

All statistical models were to be set up using general linear modeling through SPM. Initially, our goal for the analysis was to conduct a treatment vs. waitlist comparison; however due to the smaller than anticipated sample size for each group (*n* = 7 treatment, *n* = 13 waitlist), we combined scans 1 and 2 of the treatment group and scans 2 and 3 of the waitlist group to examine grey matter volume differences before and after intervention. Paired participant smoothed grey matter volumes were entered into a second level analysis using the “Flexible factorial” module in CAT12. To estimate differences in pre-post grey matter in children with DCD, a repeated measure analysis of variance (ANOVA) design with 5,000 permutations with an alpha level of 0.05 was used, with whole and within exchangeability blocks. Threshold-Free Cluster Enhancement (TFCE) thresholding was conducted using the TFCE Toolbox Version r214^2^ with 5,000 permutations (Draper–Stoneman method) with equal variance (patients) with an *E* = 0.5 and *H* = 0.2. TIV was mean-centered and used as a covariate/nuisance variable as recommended in VBM analysis to account for intra-individual differences. In order to conserve degrees of freedom, age, attentional performance, and sex were not included as covariates in this analysis. Initially, a regression analysis was proposed to examine the relationship between grey matter volume and COPM, PQRS, and BOT-2. However, PQRS and COPM were significantly positively correlated (*r* = 0.39, *p* = 0.046) for this sample (Hinkle et al., 2003). Instead, we used a regression analysis with only grey matter volume, BOT-2 (motor performance), and PQRS (actual performance quality), controlling for the effect of intracranial volume. PQRS was used instead of COPM as it is a more objective measure, despite COPM being our primary outcome. Structural images were analyzed using TFCE due to its increased sensitivity compared to voxel- or cluster-based statistics (Smith and Nichols, 2009; Salimi-Khorshidi et al., 2011; Radua et al., 2014). We assessed statistical significance with the permutation test included in SPM.

All results are reported with TFCE thresholding uncorrected for multiple comparisons (no p_FDR-corrected_ or p_FWE-corrected_) but corrected for the number of planned comparisons (pre > post, pre < post). Results are presented at *p* < 0.001 with cluster size threshold at 50 voxels. Cluster size threshold was based on current literature regarding cluster thresholding. Given our N < 50, we opted for a more stringent cluster threshold of 50 compared to lower thresholds of 10 (Lieberman and Cunningham, 2009; Woo et al., 2014). This is also comparable to previous publications of cerebellar VBM with samples of children with neurodevelopmental disabilities (D’Mello et al., 2015). Uncorrected results (*p* < 0.001) minimize false negative results but do increase the false-positive rate (Durnez et al., 2014).

## 3 Results

### 3.1 Final sample

**Figure 2:**
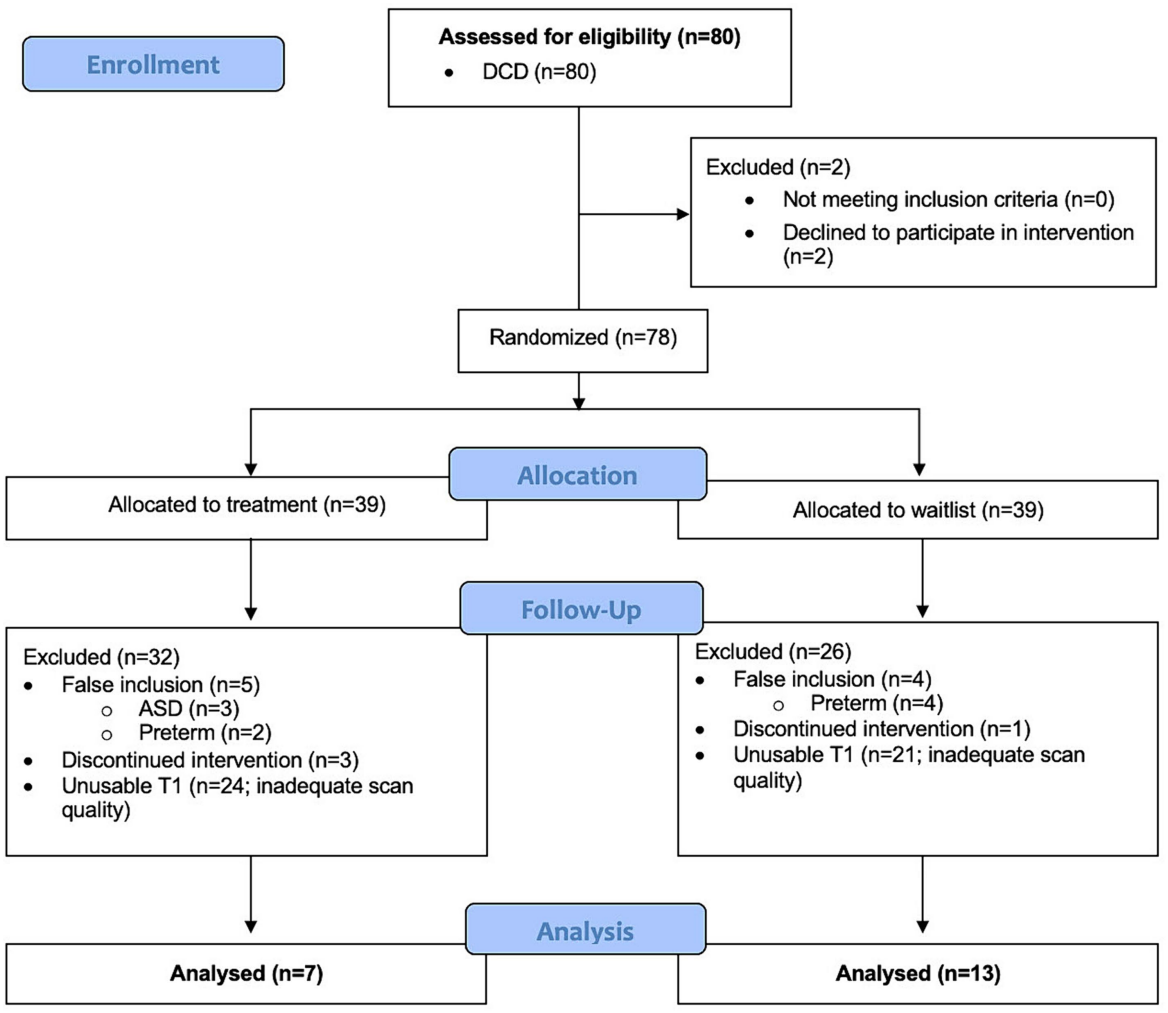
Flow diagram of participant inclusion and exclusion for longitudinal voxel-based morphometry analysis.

This study recruited 80 children with DCD+/−ADHD from which 60 were excluded because they either had co-occurring ASD or preterm birth (*n* = 9), declined to participate in the intervention (*n* = 2), discontinued intervention (*n* = 4), or had insufficient data quality for both scans (before and after intervention) to conduct VBM analysis (*n* = 45) (Figure 2). In order to preserve power, the DCD and DCD + ADHD group were combined. Our final sample for voxel-based morphometry analysis after quality checks comprised of 20 children with DCD [mean (SD) age = 9.9 (1.6) years], of which 70% were males.

Similarly, 35 TD children were initially recruited, from which 26 were excluded because they either declined to participate (*n* = 1), had ADHD (*n* = 1), were born preterm (*n* = 1), had a MABC-2 ≤16th percentile (*n* = 1), or had insufficient data quality for both scans (3 months separation) to conduct VBM analysis (*n* = 22).

No difference was found between children with DCD with good or bad quality T1 images based on BOT2 or PQRS scores. Difference in Connors *T*-score was close to significant [*p* = 0.086, CI95% = (−8.67, 0.57)]. The loss of children due to poor image quality was similar between the TD and DCD cohorts—71 and 70%, respectively—suggesting that getting quality T1 images from children this age is difficult in general.

Children with DCD whose data was excluded due to motion had an average CJV of 0.68 (±0.13 SD), while those that were kept had an average CJV of 0.61 (±0.09 SD). These values were significantly different [*p* = 0.002; CI95% = (0.03, 0.11)]. Of the participants that were included for analysis, there was no difference in CJV between the TD and DCD cohorts. Furthermore, for the children with DCD included for analysis, no correlation was found between CJV and MACB-2 scores [CI 95% = (−0.36, 0.06)].

### 3.2 Participant characteristics

Table 1 presents demographic and behavioral characteristics of the sample. Our final sample included 20 participants with DCD (Figure 2), of which 19 (95%) had attentional difficulties as indicated by a score of 70 or greater on the Conners ADHD Index. This is further supported by current literature which suggests a greater than 50% overlap between the DCD and ADHD (Kadesjö and Gillberg, 1998; Goulardins et al., 2015; Lange, 2017). Lastly, our sample of DCD included 14 males (70%), which aligns with previous literature indicating high prevalence of DCD in males (American Psychiatric Association, 2013). We also included 9 TD participants to investigate if any grey matter changes were due to maturation over a 3 months period.

**Table 1:** Description of cohort (*N* = 20).

Participant characteristics	DCD *N* (%) or mean (SD)	TD *N* (%) or mean (SD)
Male	14 (70)	4 (44)
Age at MRI (years)	9.9 (1.6)	10.4 (1.5)
MABC-2 (percentile)	5.5 (8.5)	61.7 (27.5)
Conners ADHD Index (*T*-scores)	87.0 (5.8)	57.1 (12.5)
DCDQ in suspected or indicative range	20 (100)	1 (11)
Total intracranial volume (L)	1.50 (0.18)	1.51 (0.09)

ADHD, attention deficit hyperactivity disorder; DCD, developmental coordination disorder; DCDQ, Developmental Coordination Disorder Questionnaire; L, litres; MABC-2, Movement Assessment Battery for Children-2nd ed.; SD, standard deviation.

### 3.3 Motor outcomes

The Wilcoxon Signed-Rank Test (non-parametric) was used to compare the before and after effect of CO-OP intervention. Participants showed statistically significant improvements (*p* < 0.001) in their motor goals (COPM), movement quality (PQRS), and motor skills (BOT-2) after CO-OP intervention (Table 2).

**Table 2:** Clinical outcomes before and after CO-OP intervention.

Clinical outcome measure	Pre-test mean (SD)	Post-test mean (SD)	*p*
COPM performance	3.1 (1.1)	6.3 (1.3)	<0.001
COPM satisfaction	2.5 (1.5)	7.2 (1.4)	<0.001
PQRS	3.1 (1.3)	6.2 (1.4)	<0.001
BOT-2 percentile	13.4 (11.7)	22.0 (18.4)	<0.001

BOT-2, Bruininks-Oseretsky Test of Motor Proficiency-2nd edition; CO-OP, Cognitive Orientation to daily Occupational Performance; COPM, Canadian Occupational Performance Measure; PQRS, Performance Quality Rating Scale; SD, standard deviation.

### 3.4 Grey matter volume changes following intervention

**Figure 3:**
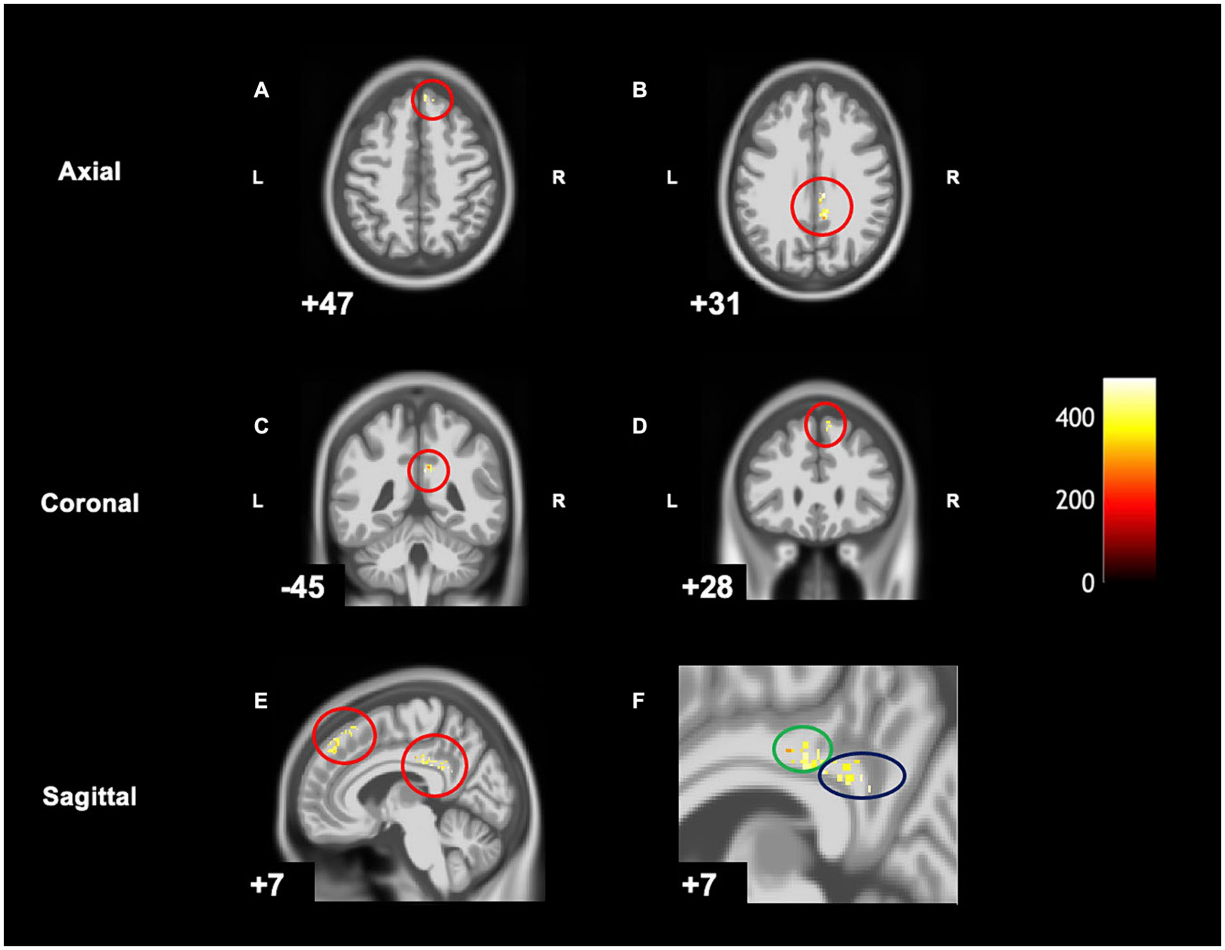
Within-group differences show significantly decreased grey matter in children with DCD after intervention on CAT T1 IXI555 GS. **(A)** Right superior frontal gyrus. **(B)** Middle and posterior cingulate gyrus. **(C)** Right middle and posterior cingulate gyrus. **(D)** Right superior frontal gyrus. **(E)** Right superior frontal gyrus, middle and posterior cingulate gyrus. **(F)** Zoomed image of **E**: middle (green) and posterior cingulate gyrus (blue).

When comparing pre-post scans, children with DCD had significantly decreased grey matter [cluster size (*k*) **>**50, *p*_uncorrected_ < 0.001] in the right hemisphere in the following regions: middle cingulate gyrus, posterior cingulate gyrus, and the superior frontal gyrus following CO-OP intervention (Table 3 and Figure 3). There were no regions where there was increased grey matter when comparing pre- intervention to post-intervention—the inverse contrast [cluster size (*k*) <50]. A follow-up analysis with age as a covariate can be found in Supplementary Figure S1.

**Table 3:** MNI coordinates for pre-post intervention differences in grey matter volume.

Location	*X*	*Y*	*Z*	TFCE	*p*_uncorrected_	Cluster size
Right middle cingulate gyrus	8	−27	31	495.2	0.001	114
Right posterior cingulate gyrus	10	−47	27	466.2	<0.001	
Right posterior cingulate gyrus	10	−38	33	463.4	<0.001	
Right superior frontal gyrus	11	24	54	494.7	<0.001	102
Right superior frontal gyrus	8	45	45	449.9	0.001	
Right superior frontal gyrus	10	37	52	445.8	<0.001	

When looking at TD children over the same period of time, no changes were found in the grey matter [cluster size (*k*) **>**50, *p*_uncorrected_ < 0.001].

### 3.5 Relationship of motor performance and performance quality to changes in grey matter volume

There were no significant positive or negative (inverse contrast) association between overall motor performance on the BOT-2 percentile scores and grey matter volume changes [cluster size (*k*) <50]. In addition, there was no significant positive or negative (inverse contrast) association between actual performance quality on motor goals after 10 weeks of intervention (as measured by PQRS scores) and grey matter volume changes [cluster size (*k*) <50].

## 4 Discussion

In this randomized waitlist-control study, we found that, following CO-OP intervention, children with DCD had decreased grey matter volume in the right superior frontal gyrus, right middle cingulate gyrus, and right posterior cingulate gyrus. We reiterate that due to sample size, we did not correct for multiple comparisons, and further work and replication of these findings is needed. The regions that showed volumetric changes are different from those identified in our previous work that investigated brain volume differences in DCD versus typically developing children using the pre-treatment scans (Malik et al., 2023). There, we showed that children with DCD had greater grey matter volume in left STG at baseline. The post-treatment findings in the right hemisphere reported here may reflect the lateralization of the brain in the early stages of learning to problem solve, or it might relate to emotional regulation (Shobe, 2014; Blais et al., 2017), both of which could be modulated via the CO-OP intervention. In other words, the intervention changes did not “normalize” the previously observed group-based or diagnostic differences in volume, though they may relate to important changes and learning functions implicated in the therapy. To contextualize and interpret the results reported in this study, we will discuss motor learning theories and background literature on the frontal and parietal regions that were found to have decreased grey matter volume.

According to general principles of motor learning and neuroplasticity, interventions that involve people in active, repetitive training can not only increase motor function (Salbach et al., 2004; Michaelsen et al., 2006; Arya et al., 2011) but can also lead to neuroplastic changes (Takeuchi and Izumi, 2013; Maier et al., 2019). Compared to TD children, children with DCD use different strategies and brain regions to improve motor performance and motor learning (Zwicker et al., 2010, 2011; Biotteau et al., 2016). Children with DCD also tend not to improve their motor skills with practice alone (Schoemaker and Smits-Engelsman, 2015), but rather benefit from using cognitive strategies and problem-solving skills to facilitate motor skill acquisition (Mandich et al., 2003; Jokić et al., 2013). CO-OP is a task-oriented intervention that combines both motor learning theories with cognitive approaches (i.e., problem-solving and self-evaluation) (Polatajko et al., 2001; Jackman et al., 2018). Additional studies underscore the self-regulation processes. For example, self-regulation is thought to be a mediator to improve motor skills (Jokić et al., 2013; Green and Payne, 2018). Brain imaging data to date seem to support this hypothesis, as brain regions associated with self-regulation showed greater structural (most findings were in default mode network) (Izadi-Najafabadi and Zwicker, 2021) and functional connectivity (default mode network and the right pregenual anterior cingulate cortex) (Izadi-Najafabadi et al., 2022b) after CO-OP intervention. Two of the brain regions that showed decreased grey matter volume after intervention, the right posterior cingulate gyrus and the right superior frontal gyrus, further support the hypothesis that changes in self-regulation may mediate improved motor function.

The posterior cingulate gyrus is one of the most densely connected regions in the brain, and has been discussed as one of the “least well-understood regions of the cortex” (Leech and Smallwood, 2019, p. 73). It functions as a hub for the default mode network (DMN), arguably one of the most complex networks in the brain (Alves et al., 2019; Buckner and DiNicola, 2019). The DMN has been implicated in a wide array of higher-order functions, most of which rely on internally constructed information (Buckner et al., 2008; Leech and Sharp, 2014; Pan et al., 2018). Perhaps the most relevant DMN functions in the current context would be network suppression during active tasks and learning combined with self-referential processing and self-regulation (Brewer et al., 2013). During CO-OP intervention, children with DCD are guided to use self-regulatory skills such as goal setting, planning, and self-monitoring to address their motor performance difficulties (Hyland and Polatajko, 2012; Jokić et al., 2013). As self-regulation is thought to mediate motor skill improvements observed with CO-OP intervention (Jokić et al., 2013; Green and Payne, 2018), it makes clinical sense that neuroplastic changes would be observed in brain regions associated with self-regulation. We propose that CO-OP intervention may reinforce synaptic connections while pruning less efficient pathways, resulting in a decrease in grey matter volume. While speculative, our interpretation is consistent with theories and findings relating to experience-dependent neuroplasticity (Giedd et al., 1999; Lenroot et al., 2007; Kleim and Jones, 2008; Wierenga et al., 2014).

The right superior frontal gyrus also showed decreased grey matter volume after CO-OP intervention. Activation of the right superior frontal gyrus is thought to modulate inhibitory control (Hu et al., 2016). Inhibitory control is defined as the suppression of behavior in response to internal or external influences (Morasch and Bell, 2011). It is a cognitive function that plays an important role in tasks such as riding a bike, where it is often necessary to prevent an action from being performed inappropriately (Coxon et al., 2007). As children with DCD have difficulty with inhibitory control of attention and executive function (Wilson et al., 1997; Wilson and Maruff, 1999; Mandich et al., 2003; Tsai, 2009; Wilson et al., 2020), impairment in inhibitory control might play a role in underlying motor coordination problems (Tsai, 2009). Our finding of decreased grey matter in the frontal region is consistent with neurodevelopmental and lesion neuroimaging studies that have identified dorsolateral and medial prefrontal cortices (responsible for unwanted response) (Rubia et al., 2005) and the right inferior frontal gyrus and basal ganglia (for cancellation of prepared or ongoing movements) (Aron et al., 2003; Chambers et al., 2006; Chikazoe et al., 2008) are involved in inhibitory control. Inhibitory control tends to improve with active intervention (Tsai, 2009), which may be associated with decreased grey matter volume in the superior frontal gyrus. As above, we speculate that this may be due to synaptic pruning of pathways that were reinforced during intervention.

The middle cingulate gyrus is known by two names: (1) dorsal anterior cingulate cortex (Palomero-Gallagher et al., 2008, 2009; Apps et al., 2013); or (2) middle cingulate cortex, which is further split into an anterior and posterior middle cingulate cortex (Apps et al., 2013; Brewer et al., 2013; Vogt, 2016). The different terminology may lead many studies to inaccurately discuss the functional properties of the middle cingulate cortex (Apps et al., 2013). Here, we adopt the more recent nomenclature of the middle cingulate gyrus, which itself is divided into dorsal, middle and posterior subsections. It has extensive connections with cognitive (e.g., lateral prefrontal) and motor-related (e.g., premotor and primary motor) areas of the cortex (Vogt and Morecraft, 2009; Stevens et al., 2011). Based on the seminal study conducted in nonhuman primates by Paus (2001), the most dorsal portion of the middle cingulate gyrus is important for the execution of voluntary motor control through its cognitive and motor connections and processing of abstract thinking and intention in motor execution. In humans, the dorsal middle cingulate gyrus is activated with motor-related tasks (Beckmann et al., 2009) and during the internal generation of movements (pre-frontal, pre-motor, parietal, basal ganglia) (Deiber et al., 1999; Cunnington et al., 2002; Debaere et al., 2003; Lau et al., 2004; van Eimeren et al., 2006; Jankowski et al., 2009). The posterior middle cingulate cortex is part of the caudal cingulate premotor area which is involved in multisensory orientation of the individual in space and in sensing the force and direction of movements in space (Vogt, 2016). Reflecting on the methodology of the intervention, to achieve the self-chosen motor goals, CO-OP uses cognitive-based strategies during task-specific intervention to facilitate motor skill acquisition. The problem-solving aspect of the intervention promotes the thought process of “what” and “how” to do a particular action. This thinking, in addition to the cognitive connections of middle cingulate gyrus may have promoted a decrease in grey matter (through increased synaptic pruning) in the middle cingulate gyrus and cognitive (posterior cingulate gyrus) and motor regions (superior frontal gyrus) found in this study.

Previously, we reported that children with DCD had greater grey matter volume in the left superior frontal gyrus compared to typically developing children (Malik et al., 2023). This is posited to be a result of aberrant brain maturation, specifically a delay or dysfunction in cortical thinning through the mechanisms (synaptic pruning) described earlier. It is thought that similar to other neurodevelopmental disorders, dysfunction of synaptic pruning may result in increased grey matter as the neural connections are not maturing as expected. This leads to difficulties in working memory and higher order cognitive functions. After having undergone 10 weeks of CO-OP intervention, the decrease in grey matter volume in the right middle and posterior cingulate gyrus and right superior frontal gyrus may be a result of increased synaptic pruning.

The short form of BOT-2 percentile (measure of motor performance) significantly increased after intervention with this sub-set of participants; however, this was not the case in the larger sample (Izadi-Najafabadi et al., 2022a). We did not see any association between grey matter changes and this measure of overall motor performance in this small sample. One possible explanation for this has to do with the nature of the intervention and neuroplasticity. CO-OP is a task-oriented intervention that focuses on using a problem-solving based framework to acquire specific motor skills; it does not address the underlying motor impairment (Polatajko et al., 2001; Blank et al., 2012; Smits-Engelsman et al., 2012). Given the principles of neuroplasticity (Kleim and Jones, 2008), we would only expect to see brain changes associated with the specific target actions, not overall motor performance; thus, it is not surprising that we did not observe a relationship between grey matter volume changes and BOT-2 scores. However, we also investigated the association between PQRS and grey matter volume changes in the brain. The PQRS is a measure of actual performance quality for the child-chosen goals that were addressed in therapy over 10 weeks. We also did not find a relationship between movement quality and grey matter volume. Given our small sample size, we are likely under-powered to detect this expected relationship.

Several limitations are present in this study. First, our sample size was limited. Of the 80 participants scanned, several scans were lost due to insufficient quality for VBM analysis at either or both time points. After stringent quality checks and exclusion criteria, the final sample was small (*N* = 20). As mentioned by Sáenz et al. (2020), this may lead to a biased sample as the significant head motion can be associated with clinical traits and the scans of the most severely impaired participants therefore might have been excluded; the exclusion of poor-quality scans also limits the generalizability of results (Sáenz et al., 2020). Relatedly, we did not correct for multiple comparisons in the analyses, and thus the results are potentially more vulnerable to false positives. The fact that the results are sensible (i.e., the identified brain regions relate to key aspects of the intervention) suggests the results may be valid, but replication is needed. Second, there are no standardized quantification guides to measure degree of motion artifact in T1 scans. We used visual inspection by two trained, independent raters based on established guidelines (Harvard Center for Brain Science, 2014), ensuring that only high-quality scans that were deemed acceptable by both raters were included. Third, while we intended to analyze children with DCD and DCD + ADHD separately, our sample for VBM analysis was smaller than expected and included children with DCD and co-occurring ADHD (*n* = 6) could have confounded our findings. However, the sample had similar Connors ADHD Index scores, suggesting the children with diagnosed ADHD were more similar than different to the DCD group. We were also unable to compare the treatment group with the waitlist group, which would have controlled for maturation, nor were we able to examine follow-up effects in the treatment group. Fourth, we did not control for age, sex, or medications, in order to conserve power. Finally, there are some limitations regarding volume-based measures. Since grey matter includes surface area and thickness, each of which have their own developmental trajectories, the interpretation of grey matter volume becomes difficult without examining surface area or thickness individually (Frye et al., 2010).

Future studies should continue to explore intervention-induced changes in grey matter volume in children with DCD but in a larger sample of children and measured overtime to see if these changes are maintained. Exploring cortical thickness and volume in one study longitudinally would provide more insight into the brain’s structural morphology in this disorder. Likewise, results could be stratified by age, sex and/or medication use to provide further insights (Caviness et al., 1996; De Bellis et al., 2001; Spencer et al., 2013). Lastly, to further explore synaptic pruning, molecular, and animal model studies should be conducted to examine the long-term intervention-induced changes in synaptic pruning in this population.

Overall, CO-OP is one of the recommended task-oriented rehabilitation interventions in the international clinical practice guidelines for DCD (Blank et al., 2019). This intervention is effective in improving children’s perceptions of their motor performance of the specific skills they wanted to learn, as well as improved motor quality while performing these skills as rated by a therapist. In conclusion, our data indicate that CO-OP induces neuroplasticity of the grey matter in the right superior frontal gyrus (inhibitory control), right posterior cingulate gyrus (self-regulation), and right middle cingulate gyrus (voluntary thinking and cognitive and motor connections) in children with DCD. We speculate that these neuroplastic changes result from upregulated synaptic pruning that occurs within the repeated actions and learning of the intervention, leading to the maturation of synapses in DCD-related circuits. This study further supports our team’s findings on how CO-OP induces changes in structure and function of brain regions associated with self-regulation, providing initial evidence for the brain-based impact of this intervention.

## Data availability statement

The raw data supporting the conclusions of this article will be made available by the authors, without undue reservation.

## Ethics statement

The studies involving humans were approved by Children’s and Women’s Health Centre/University of British Columbia. The studies were conducted in accordance with the local legislation and institutional requirements. Parents/legal guardians provided informed written consent and children assented to participate in the study.

## Author contributions

MM: Data curation, Formal analysis, Investigation, Visualization, Writing – original draft. DL: Formal analysis, Writing – review & editing. TV: Formal analysis, Writing – review & editing. JZ: Conceptualization, Formal analysis, Funding acquisition, Methodology, Project administration, Resources, Supervision, Visualization, Writing – review & editing. AW: Formal analysis, Visualization, Writing – review & editing.

## Footnotes

^1^ https://www.nitrc.org/projects/mricron

^2^ http://dbm.neuro.uni-jena.de/tfce/
