# Supplementary material for: Changes in cortical grey matter volume with Cognitive Orientation to daily Occupational Performance intervention in children with developmental coordination disorder
Source: Front Hum Neurosci. 2024 May 22;18:1316117. doi: 10.3389/fnhum.2024.1316117 (PMC11150831; doi:10.3389/fnhum.2024.1316117)
Supplement: Supplementary file 1 [file Table_1.DOCX]

Supplementary Materials

For our study, it was important to consider age-related changes in cortical grey matter volume, particularly in pediatric populations where developmental trajectories can significantly impact neuroimaging outcomes. Therefore, a follow-up analysis with age as a covariate was also performed. The clusters of significance that were discovered and reported from our original analysis remained. Indeed, including age as a covariate increased the number of clusters of significance, suggesting that changes due to CO-OP may have actually been masked by maturation effects, and that CO-OP has more effect than we initially found. However, we remain concerned that by adding age into the analysis, we may be introducing false positives. As adding a covariate can increase the complexity of a statistical model and introduce more variability into the analysis, we believe this may be the case here (Supplementary Figure S1).

**
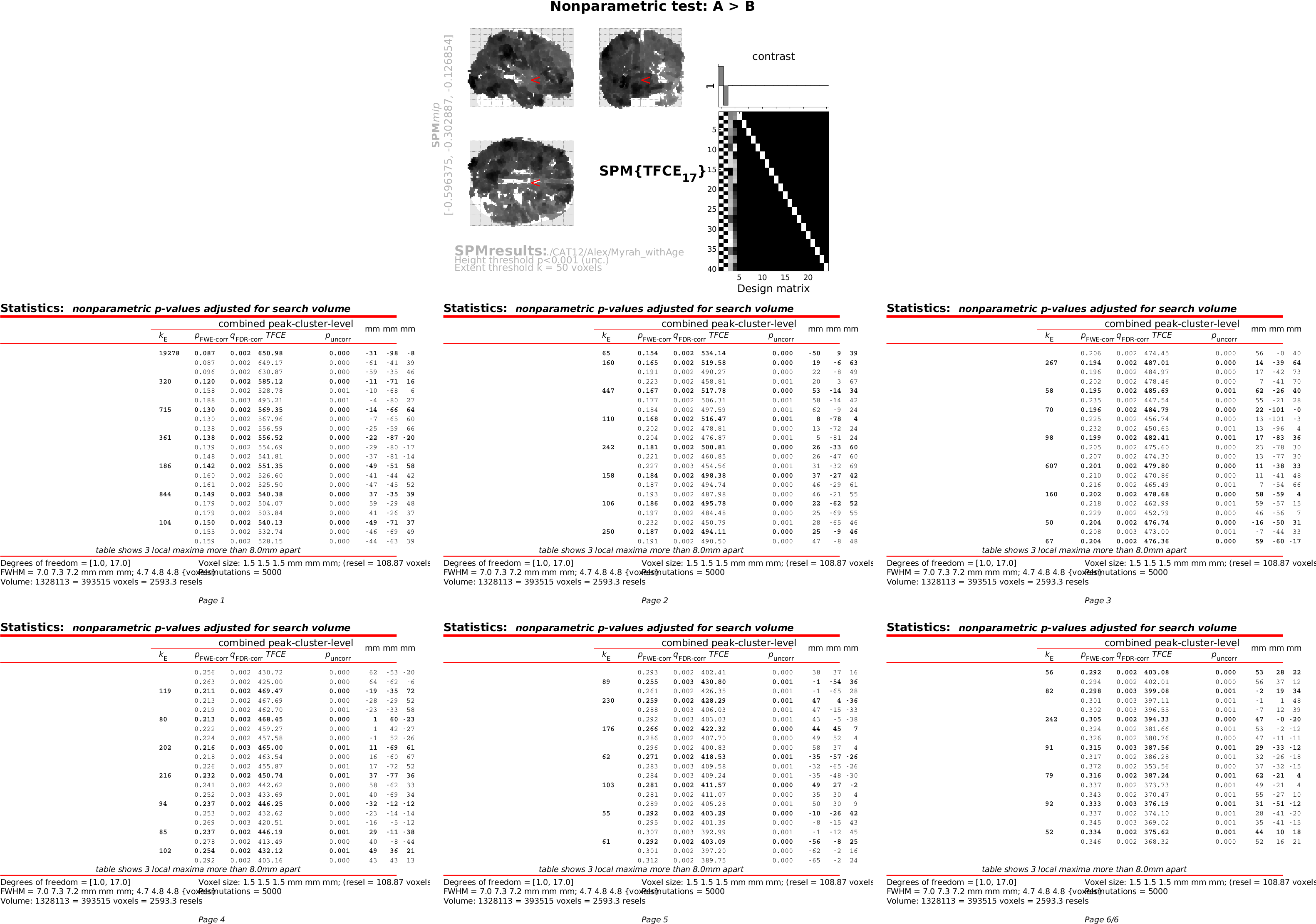
Supplementary Figure S****1: Regions of within-group differences showing decreased grey matter volume in children with DCD after intervention when including age as a covariate.** This figure is taken directly from the CAT12 TFCE results output. The top-centre image shows a minimum intensity projection of areas with clusters of significance (before treatment > after treatment) in sagittal, coronal and axial planes. The design contrast matrix can be seen just to the right of these, with TIV and age as covariates. Six tables showing clusters of significance, including the cluster size, p-values, and location, are included (middle and bottom).
